# Latent class profiles of health-risk behaviors and the protective roles of psychological resilience and self-control in Chinese adolescents

**DOI:** 10.3389/fpubh.2026.1908835

**Published:** 2026-08-19

**Authors:** Lvhao Liu, Jiajia Chen, Yongsheng Wang, Tao Xu

**Affiliations:** 1 School of Physical Education and Health, Guangxi Normal University, Guangxi, China; 2 College of Sports Science, Jishou University, Jishou, China

**Keywords:** adolescent, health-risk behaviors, latent class analysis, psychological resilience, self-control

## Abstract

**Background:**

Health-risk behaviors (HRBs) frequently co-occur during adolescence and are associated with adverse physical and mental health outcomes. This study aimed to identify latent classes of HRBs and examine the associations of psychological resilience (PR) and self-control (SC) with class membership.

**Methods:**

In this cross-sectional study conducted in 2023, 11,407 students from 90 schools were recruited using multistage stratified cluster sampling. Latent class analysis (LCA) was used to identify patterns of HRBs. Chi-square tests and multinomial logistic regression were used to examine sociodemographic and psychological factors associated with class membership.

**Results:**

Four latent classes were identified: the Low-Risk Group (45.6%), the Physical Inactivity–Insufficient Sleep Group (21.8%), the Depressive Symptoms–Self-Harm–Picky Eating Group (26.3%), and the Multiple Health-Risk Behavior Group (6.4%). Relative to the Low-Risk Group, each one-point increase in psychological resilience was associated with a 3.4% reduction in the odds of membership in the Physical Inactivity–Insufficient Sleep Group (OR = 0.966, 95% CI: 0.957–0.976), a 6.6% reduction in the Depressive Symptoms–Self-Harm–Picky Eating Group (OR = 0.934, 95% CI: 0.925–0.942), and a 4.2% reduction in the Multiple Health-Risk Behavior Group (OR = 0.958, 95% CI: 0.945–0.972). Corresponding reductions in odds for self-control were 2.1% (OR = 0.979, 95% CI: 0.973–0.986), 6.2% (OR = 0.938, 95% CI: 0.932–0.943), and 8.8% (OR = 0.912, 95% CI: 0.903–0.921). Male gender was associated with significantly elevated odds of membership in the Multiple Health-Risk Behavior Group (OR = 2.275, 95% CI: 1.808–2.863), whereas rural residence was associated with lower odds of membership in the Physical Inactivity–Insufficient Sleep Group (OR = 0.530, 95% CI: 0.452–0.620) and the Depressive Symptoms–Self-Harm–Picky Eating Group (OR = 0.645, 95% CI: 0.565–0.737).

**Conclusion:**

Chinese adolescents exhibited heterogeneous patterns of HRBs. Higher psychological resilience and self-control were independently associated with lower odds of membership in the three non-low-risk classes. These findings may inform class-specific prevention strategies.

## Introduction

Adolescence is a period full of opportunities and challenges. Teenagers have the chance to learn new things in life, but compared to adults, they are more likely to engage in risky behaviors, which can lead to negative consequences (1). These behaviors are described as health-risk behaviors (HRBs). Adolescence is an important stage of personal growth. Risky health behaviors during this period can affect both physical and mental health and may also pose risks to health in adulthood (2, 3). Health-risk behaviors include lack of exercise, insufficient sleep, an unbalanced diet, smoking, excessive drinking, risky sexual behaviors, and adverse experiences (4). These behaviors are common among adolescents and cause significant harm to themselves and potentially to others they come into contact with (5–7). The incidence of risky behaviors among adolescents is highly correlated with their mortality rate (8). UNICEF reported that approximately 70% of adolescent premature deaths are attributable to HRBs that originate during puberty (9). In addition, health-risk behaviors among adolescents usually do not occur in isolation; multiple behaviors often coexist, such as smoking, drinking, lack of exercise, and poor nutrition, which are gradually drawing people’s attention (10, 11). This clustering is of great significance for research and practice, because certain behaviors share similar characteristics, and therefore, changing one behavior can affect the prevalence of another (12). At the same time, interventions targeting clustered health behaviors have been shown to be more effective and cost-efficient (13).

Previous studies have analyzed various categories of health-risk behaviors in adolescents (14–16), Ignored the issue of heterogeneity within the group.person-centered approach(PCA)It is possible to divide individuals into subgroups with relatively high internal homogeneity, which facilitates comparative analysis of different subgroups from both quantitative and qualitative dimensions. PCA Common data analysis strategies include latent class analysis (LCA), latent profile analysis (LPA)and latent transition analysis (LTA) (17). LCA can identify unobservable subgroups within a population, thereby better understanding the exposure effects of multiple risk patterns and the antecedents and consequences of complex behaviors, in order to develop targeted interventions to help the subgroups most likely to benefit (18). In addition, an individual-centered approach is used to describe how patterns of risky behavior change over time (19, 20). A latent class study of 22,628 adolescents in six Chinese cities assessed six risky behaviors (non-suicidal self-injury in the past year, suicidal behavior in the past year, accidental injuries in the past year, smoking and alcohol use in the past 30 days, and daily screen time); latent class analysis (LCA) identified four classes: “low-risk group,” “smoking, alcohol use, and screen time group,” “NSSI, suicidal behavior, and unintentional injury group,” and “high-risk group” (21).

Psychological resilience is a positive mental quality that reflects an individual’s ability to cope with and overcome adversity (22). There is a significant association between psychological resilience and various harmful health behaviors. Individuals with strong psychological resilience have a strong adaptive capacity to actively cope with the negative impacts of adversity, thereby reducing the stress caused by negative emotions and improving their psychological wellbeing. Moreover, existing research has shown that good psychological resilience can help individuals maintain healthy and stable mental states (23). During adolescence, experiences of childhood adversity can reduce psychological resilience, thereby increasing the risk of various health-harming behaviors, including externalizing behaviors (such as aggression and substance abuse) and internalizing behaviors (such as depression and anxiety) (24). Psychological resilience is also related to self-control ability, and higher psychological resilience helps individuals maintain healthy behaviors and a good lifestyle under stress (25). Self-control refers to an individual’s ability to inhibit impulses, resist temptations, delay gratification, and maintain goal-directed behavior. It is an important concept in psychology for explaining differences in healthy and unhealthy behaviors (26–28). High self-control is positively associated with healthy behaviors (such as regular exercise and healthy eating), while low self-control is closely related to various health-harming behaviors (such as excessive drinking, smoking, overeating, sedentary lifestyle, bullying, etc.). Moreover, the lower the self-control, the higher the risk of clustering of health-harming behaviors (26–30). Recent studies have also revealed that self-control not only directly affects health behaviors but also indirectly influences health outcomes through social cognition, intentions, coping methods, and other factors (27, 31). Lack of self-control is considered a core mechanism behind the failure of healthy behaviors, involving multiple factors such as cognitive, emotional, and social aspects (32). Therefore, enhancing psychological resilience and self-control, reducing external stress, and optimizing coping strategies are important directions for preventing and intervening in health-risk behaviors.

At the family level, the development of risky health behaviors in adolescents can sharply increase family tension and rapidly deteriorate parent–child communication. At the school and societal levels, the development of health-risk behaviors can have a negative impact on students’ academic performance (33), Frequent incidents of fighting, smoking, and drug abuse among teenagers on campus seriously disrupt the teaching order and increase the burden on teachers (34), Involvement in risky behaviors during adolescence is not only a major cause of abnormal deaths among teenagers, but also increases their risk of imprisonment for crimes in adulthood, causing significant losses to society (35). Adolescent health-risk behaviors not only harm individual health but also spread externally through a three-level pathway of “family burden—school resource strain—public health and safety costs,” already constituting sources of family stress, challenges for school management, and public health issues in society.

China has the largest youth population in the world, with over 200 million primary and secondary school students, accounting for 20% of the total population (36). In China, adolescent health-risk behaviors have attracted public attention, but there are few studies reporting on LCA of health-risk behaviors, and no scholars have examined the relationship between adolescent health-risk behavior LCA and psychological resilience and self-control. This study uses four categories of behaviors—deliberate injury, substance addiction, psychological addiction, and unhealthy eating—as a framework to investigate nine common health-risk behaviors among adolescents. A heterogeneous classification of health-risk behaviors was conducted among 11,407 Chinese adolescent students to construct an LCA category model, explore the potential categories of students’ health-risk behaviors, further investigate the factors influencing health-risk behaviors among students of different latent categories, and examine how adolescent psychological resilience and self-control can reduce the risk of health-risk behaviors.

## Methods

### Study design and participants

#### Sample

A multi-stage stratified cluster sampling method was used to select research subjects. In the first stage, based on geographic location, Guangdong, Shandong, and Zhejiang provinces were selected in eastern China; Hunan, Hubei, and Henan provinces in central China; and Guangxi, Chongqing, and Guizhou provinces in western China, totaling nine provinces. In the second stage, four junior high schools, four senior high schools, and two universities were randomly selected in each province, totaling 90 schools. In the third stage, using grade as the stratification criterion, one class was randomly selected from each grade level: grades 7–9 in junior high school, grades 10–12 in senior high school, and years 1–4 in university, totaling 288 classes. Students with intellectual disabilities were excluded, and all students meeting the criteria were included as study subjects. A total of 12,063 participants were ultimately recruited for the survey. After excluding 656 invalid questionnaires, 11,407 valid questionnaires were obtained, resulting in a valid response rate = 94.6%. Questionnaires were defined as invalid if they met any of the following criteria: (1) extremely short completion time indicating careless responses; (2) monotonous or patterned answers across all rating items; (3) over 30% missing values on core health-risk behavior indicators; (4) logical contradictions in key items. The participants were adolescents with a mean age of 15.6 ± 2.8 years, including 5,605 junior high school students, 2,524 senior high school students, and 3,278 university students. The university sample was predominantly composed of freshmen (18 years) and sophomores (19 years), with only a small proportion of upper-year students. All participants signed informed consent forms, and for participants under 18 years old, parental informed consent was also obtained. This survey was approved by the Medical Ethics Committee of Jishou University (Approval No: JSDX-2023-0011).

### Measures

#### Health-risk behaviors

This survey was conducted using the “Questionnaire on Health-Risk Behaviors of Adolescents,” which was developed based on Ji Chengye’s “Comprehensive Report on Health-Related/Risk Behaviors of Chinese Adolescents, 2005” (37). This questionnaire is structured around four types of behaviors: deliberate injury, substance addiction, mental addiction, and unhealthy eating, and examines nine common health-threatening behaviors among adolescents. The nine health-threatening behaviors selected for this study include smoking, drinking, picky eating, insufficient sleep, lack of physical activity, violent behavior, self-harm, smartphone addiction, and depressive symptoms. Among these, seven behaviors—smoking, drinking, picky eating, insufficient sleep, lack of physical activity, violent behavior, and self-harm—were measured using items from the “Comprehensive Report on Health-Related/Risky Behaviors of Chinese Adolescents 2005,” written by Ji Chengye and published by Peking University Medical Press.

#### Smoking

The criterion for determining smoking is defined as having inhaled and exhaled one or more tobacco-containing products (including cigarettes, cigars, hookah, e-cigarettes, etc.) on any day within the past 30 days.

#### Drinking alcohol

The criterion for determining alcohol consumption refers to having consumed one or more alcoholic beverages (ethanol content ≥0.5%) on any day in the past 30 days, regardless of the amount consumed in a single instance or the drinking occasion.

#### Picky eating

The criteria for determining picky eating refer to a persistent aversion to one or more types of food (such as fruits, vegetables, meat, dairy products, grains, and tubers), leading to intentional avoidance of consumption, and this avoidance behavior has resulted in a monotonous diet or restricted sources of nutrients.

#### Insufficient sleep

The criteria for insufficient sleep refer to experiencing difficulty falling asleep, waking up during the night, or waking up early “often” or “always” due to emotional worry or anxiety within the past 12 months, resulting in a total subjective or objective sleep duration below the recommended amount for the same age group.

#### Physical inactivity

The criterion for physical inactivity refers to the number of days in the past week that the respondent exercised for 60 min each day. If participants exercised for 60 min on fewer than 3 days per week, they were classified as having insufficient physical activity.

#### Violent behavior

The criteria for determining violent behavior refer to whether the respondent has had at least one physical conflict with others in the past 12 months that resulted in injury to themselves or others.

#### Self-harm behavior

The criteria for determining self-injurious behavior refer to intentionally inflicting physical harm on oneself at least once in the past 12 months for the purpose of emotional regulation, without suicidal intent. Forms of such behavior include, but are not limited to, cutting, burning, biting, pinching, pulling hair, and hitting hard objects.

#### Depressive symptoms

Depressive symptoms were assessed using the Patient Health Questionnaire-9 (PHQ-9) (38) A survey was conducted using this scale, which is based on the diagnostic criteria for depressive symptoms in the American “Diagnostic and Statistical Manual of Mental Disorders, Fourth Edition” (DSM-IV). The PHQ-9 has been validated for use among adolescents, including Chinese secondary school students aged 11–19 years, and has demonstrated favorable psychometric properties for assessing depressive symptoms in this population (39). The results of this survey showed that the internal consistency reliability of the scale (Cronbach’s *α*) was 0.88, the split-half reliability was 0.88, and the test–retest reliability over a 14-day interval was 0.85 (*p* < 0.05), indicating that the measurement tool is reliable. The criteria for depressive symptoms were defined as a score greater than 4 on the PHQ-9 questionnaire, which is considered indicative of the presence of depressive symptoms (40).

#### Smartphone addiction

The MPAI consists of 17 items (total score 17–85); smartphone addiction was defined as total score ≥ 34 based on Chinese adolescent norms (41). This scale consists of 17 items, divided into four dimensions: withdrawal, loss of control, inefficiency, and avoidance. Reliability tests showed that for the total scale, Cronbach’s *α* coefficient and split-half reliability coefficient were 0.91 and 0.87, respectively, and the test–retest reliability (interval of 14 days) was 0.81 (*p* < 0.05). At the dimension level, the Cronbach’s α coefficients for the four dimensions were 0.80, 0.84, 0.82, and 0.91; the split-half reliability coefficients were 0.83, 0.76, 0.78, and 0.90, respectively. These data indicate that the scale has ideal reliability indicators.

#### Psychological resilience

Psychological resilience was assessed using the 10-item Connor–Davidson Resilience Scale (CD-RISC-10) (42). This scale was originally developed by Connor-Davidson and included 25 items. It was later simplified by Campbell-Stills and Stein, resulting in the CD-RISC10. The Chinese version was translated and revised by Wang Li and others (43), Widely used in China to measure individuals’ levels of psychological resilience, this scale has a Chinese version consisting of 10 items and uses a 5-point Likert scoring method, where 0 represents “never,” 1 represents “rarely,” 2 represents “sometimes,” 3 represents “often,” and 4 represents “always.” The total score is the sum of the scores for each item, and a higher score indicates a higher level of psychological resilience. The scale demonstrates good reliability and validity, with a Cronbach’s *α* coefficient of 0.91. In this study, the Cronbach’s α coefficient was 0.90, and the split-half reliability was 0.86.

#### Self-control

Compiled by Tangney et al. (44). The Self-Control Scale (SCS) revised by Tan Shuhua and Guo Yongyu consists of 19 items, covering five dimensions: impulse control, healthy habits, resisting temptation, focusing on work, and moderating entertainment. It uses a 5-point rating scale, ranging from “1 = does not apply at all” to “5 = applies very much,” with scores ranging from 19 to 95. Higher scores indicate stronger self-control ability. In this study, the Cronbach’s *α* coefficient of this scale was 0.81, and the split-half reliability coefficient was 0.79.

### Statistical analysis

A database was established using EpiData 3.1, and data were entered using double-entry. Latent class analysis was conducted using Mplus 8.3 software, with nine health-risk behavior indicators. Participants were assigned to the latent class for which they had the highest posterior probability. Given the good classification accuracy of the retained four-class model (entropy = 0.889), most-likely class membership was treated as an observed categorical outcome in the subsequent multinomial logistic regression analyses. Measurement data are expressed as mean ± standard deviation. Data processing and analysis were performed using SPSS 27.0 software. The χ^2^ test was used to compare categorical variables across latent classes, whereas one-way analysis of variance was used to compare psychological resilience and self-control scores. Additionally, χ^2^ tests were used to compare the prevalence of individual health-risk behaviors across educational stages. Multinomial logistic regression was used to examine factors associated with latent class membership. No missing values were present in the variables included in the analyses. Therefore, the latent class analyses, the main-effects multinomial logistic regression model, and all interaction models were conducted using the full analytical sample of 11,407 participants. Multivariable multinomial logistic regression was applied to explore factors associated with latent class membership. Based on the social-ecological theoretical framework and univariate analysis results, multiple demographic, familial and psychological variables were simultaneously included in the fully adjusted models. Covariates included gender, residence type, educational stage, left-behind status, family structure, paternal education, only-child status, psychological resilience, and self-control. Maternal education was excluded because of its strong association with paternal education (Phi coefficient > 0.80). The Low-Risk Group was set as the reference category. Multinomial logistic regression was adopted to examine the associations between psychological resilience, self-control, and latent class membership, using the Low-Risk Group as the reference category. Odds ratios (ORs) and 95% confidence intervals (CIs) were computed to assess the strength of the associations. To examine whether the associations of psychological resilience and self-control with latent class membership varied across subgroups, interaction terms between psychological resilience/self-control and demographic variables (sex, educational stage, and family structure) were tested in separate models. Psychological resilience and self-control were mean-centered before the interaction terms were constructed. Sex was coded as female = 0 and male = 1; educational stage was coded as university = 0, junior high school = 1, and senior high school = 2; and family structure was coded as two-parent household = 0 and single-parent household = 1. All interaction models used the Low-Risk Group as the outcome reference category and were adjusted for the same covariates as the main-effects model. Table 1 presents the adjusted main-effect estimates, whereas Table 2 presents only the interaction terms. A two-tailed *p* < 0.05 was considered statistically significant. All statistical analyses were conducted using the unweighted observed sample data. Accordingly, the frequencies and percentages reported in Tables 3, 4 represent unweighted observed values.

**Table 1:** Multiple logistic regression predicts potential category membership.

Variables	Depressive symptoms–self-harm–picky eating		Physical inactivity–insufficient sleep		Multiple health-risk behavior	
	*P*	OR(95%CI)	*P*	OR(95%CI)	*P*	OR(95%CI)
Gender						
Male	0.000	0.465(0.412–0.525)	0.000	0.408(0.354–0.469)	0.000	2.275(1.808–2.863)
Female						
Residence type						
Rural	0.000	0.645(0.565–0.737)	0.000	0.530(0.452–0.620)	0.001	0.681(0.540–0.859)
Urban						
Left-behind child						
Yes	0.539	1.045(0.908–1.203)	0.657	1.038(0.882–1.222)	0.767	1.036(0.818–1.313)
No						
Family structure						
two-parent household	0.057	1.162(0.996–1.356)	0.801	0.977(0.813–1.173)	0.846	1.025(0.798–1.318)
Single-parent household						
Psychological resilience	0.000	0.934 (0.925–0.942)	0.000	0.966(0.957–0.976)	0.000	0.958(0.945–0.972)
Self-control	0.000	0.938 (0.932–0.943)	0.000	0.979(0.973–0.986)	0.000	0.912 (0.903–0.921)
Educational stage						
Junior high school	0.000	4.763 (4.087–5.552)	0.000	0.323 (0.289–0.361)	0.274	1.113 (0.919–1.349)
Senior high school	0.000	8.063 (6.813–9.541)	0.000	0.769 (0.674–0.878)	0.000	2.137 (1.721–2.652)
University						
Paternal education						
High education	0.052	1.103 (0.999–1.219)	0.039	1.120 (1.006–1.248)	0.508	0.943 (0.792–1.123)
Low/middle education						
Only child status						
Only child	0.706	0.976 (0.859–1.109)	0.243	0.927 (0.816–1.053)	0.000	1.447 (1.198–1.747)
Non-only child						

Multinomial logistic regression was conducted with the Low-Risk Group as the outcome reference category. Reference categories were female for sex, urban for residence, university for educational stage, single-parent household for family structure, low/middle paternal education, non-only child, and non-left-behind child. The model included only main effects and was adjusted for all variables shown in the table. OR, odds ratio; CI, confidence interval.

**Table 2:** Interaction effects of psychological resilience and self-control on latent class membership.

Interaction terms	Physical inactivity–insufficient sleep OR (95%CI)	Depressive symptoms–self-harm–picky eating OR (95%CI)	Multiple health-risk behavior OR (95%CI)
Resilience × Sex	1.034(1.014–1.054)^**^	1.033(1.016–1.050)^***^	0.971(0.943–0.999)^*^
Self-control × Sex	0.995(0.982–1.009)	1.009(0.997–1.021)	0.994(0.974–1.015)
Resilience × Educational stage	1.105(0.823–1.485)	1.610(1.246–2.080)^**^	1.865(1.156–3.008)^*^
Self-control × Educational stage	0.995(0.982–1.009)	1.007(0.994–1.020)	0.979(0.961–0.998)^*^
Resilience × Family structure	1.007(0.982–1.031)	1.012(0.992–1.032)	0.999(0.971–1.027)
Self-control × Family structure	0.994(0.976–1.011)	0.996(0.981–1.011)	1.008(0.987–1.029)

In this table reports only the interaction terms; adjusted main-effect estimates are presented in Table 1. The Low-Risk Group was used as the outcome reference category. Each interaction was tested in a separate multivariable multinomial logistic regression model. OR, odds ratio; CI, confidence interval. ***P < 0.001, **p < 0.01, *p < 0.05.

**Table 3:** Descriptive characteristics of the participants (*n* = 11,407).

Variables	*n* (%) or mean ± SD
Health risk behaviors	
Smoking	850(7.5)
Drinking alcohol	1,104(9.7)
Smartphone addiction	3,117(27.3)
Picky eating	3,804(33.3)
Insufficient sleep	6,440(56.5)
Physical inactivity	6,208(54.4)
Depressive symptoms	3,092(27.1)
Violent behavior	1859(16.3)
Self-harm behavior	2,161(18.9)
Individual level	
Male	5,477(48.0)
Female	5,930(52.0)
Rural	4,351(38.1)
Urban	7,056(61.9)
School level	
Junior high school	5,605(49.1)
Senior high school	2,524(22.1)
University	3,278(28.8)
Family level	
Paternal education	
Primary or below	3,560(31.2)
Junior high or above	7,847(68.8)
Maternal education	
Primary or below	2,947(25.8)
Junior high or above	8,460(74.2)
two-parent household	9,359(82.0)
Only child	2045(17.9)
Left-behind children	3,258(28.6)
Psychological level	
Self-control	60.2 ± 11.2
Psychological resilience	33.3 ± 8.3

Data are presented as unweighted n (%) or mean ± standard deviation (SD).

**Table 4:** Univariate analysis of latent class characteristics of health risk behaviors in Chinese adolescents.

Variables	Low-risk	Depressive symptoms–self-harm–picky eating	Physical inactivity–insufficient sleep	Multiple health-risk behavior	*X* ^2^ */F*	*P*
Gender					755.908	0.000
Male	2,919 (53.3)	1,177 (21.5)	806 (14.7)	575(10.5)		
Female	2,280 (38.4)	1821 (30.7)	1,679(28.3)	150 (2.5)		
Educational stage					1438.105	0.000
Junior high	2,741 (48.9)	1823 (32.5)	707 (12.6)	334 (6.0)		
Senior high	848 (33.6)	953 (37.8)	518 (20.5)	205 (8.1)		
University	1,610 (49.1)	222 (6.8)	1,260 (38.4)	186 (5.7)		
Residence type					179.696	0.000
Rural	2,191 (50.4)	863 (19.8)	1,063(24.4)	234 (5.4)		
Urban	3,008 (42.6)	2,135 (30.3)	1,422(20.2)	491 (7.0)		
Only child					31.560	0.000
Yes	925 (45.2)	472 (23.1)	472 (23.1)	176 (8.6)		
No	4,274 (45.7)	2,526 (27.0)	2013 (21.5)	549 (5.9)		
Left-behind child					9.372	0.025
Yes	1,468 (45.1)	813 (25.0)	764 (23.4)	213 (6.5)		
No	3,731 (45.8)	2,185 (26.8)	1721 (21.1)	512 (6.3)		
Paternal education					22.047	0.000
Primary or below	1,559 (43.8)	1,035 (29.1)	760 (21.3)	206 (5.8)		
Junior high or above	3,640 (46.4)	1963 (25.0)	1725 (22.0)	519 (6.6)		
Maternal education					40.454	0.000
Primary or below	1,272 (43.2)	769 (26.1)	754 (25.6)	152 (5.2)		
Junior high or above	3,927 (46.4)	2,229 (26.3)	1731(20.5)	573 (6.8)		
Family structure					43.545	0.000
two-parent household	4,355 (46.5)	2,361 (25.2)	2076(22.2)	567 (6.0)		
single-parent household	844 (41.2)	637 (31.1)	409 (20.0)	158 (7.7)		
Psychological resilience	36.1 ± 7.8	29.1 ± 7.5	33.0 ± 7.5	31.3 ± 9.6	424.132	0.000
Self-control	63.7 ± 10.8	55.7 ± 10.1	60.7 ± 10.1	52.6 ± 11.9	390.936	0.000

Values are presented as unweighted n (%) or mean ± standard deviation (SD).

LCA is a statistical method used to classify individuals and identify group heterogeneity based on differences in individuals’ response patterns to categorical observed variables (45). This method categorizes individuals into several latent classes by identifying inherent similarities in their response patterns, aiming to minimize differences within each class while maximizing differences between classes. This goes beyond the coarse classification based on overall scores and can more accurately reveal the heterogeneity of the population. LCA is a type of latent variable analysis. To distinguish it from latent variables, the observable explicit variables are called indicators. In LCA, both the latent variables and their indicators are categorical variables (46). The statistics used to evaluate or test the goodness of fit of models mainly include the Pearson chi-square and the likelihood ratio chi-square, as well as information criteria based on the log-likelihood (LL) value, such as AIC (Akaike Information Criterion), BIC (Bayesian Information Criterion), aBIC (adjusted Bayesian Information Criterion), and also entropy (46). Among them, AIC, BIC, and aBIC represent model fit, and lower values indicate a better model fit (47). Information entropy (Entropy) has a range of 0–1. The closer the value is to 1, the more accurate the classification. When the information entropy is greater than 0.8, it indicates that the classification accuracy exceeds 90% (48) If the *p*-values of the LMR and Bootstrap-based likelihood ratio test (BLRT) indicators reach a significant level, it indicates that the model with k classes is significantly better than the model with k-1 classes (49). To examine whether the latent class structure differed across educational stages, additional stratified latent class analyses were conducted separately among junior high school students, senior high school students, and university students. For each educational-stage subgroup, models with two to five latent classes were estimated using the same nine binary health-risk behavior indicators and the same model-selection criteria as those used in the full-sample analysis. Detailed model-fit indices and class-specific conditional probabilities are presented in Supplementary material.

## Results

### Descriptive statistics

Table 3 describes the sociodemographic variables of the study. In the study sample, females accounted for the majority (52%), and more than half of the participants came from urban areas (61.9%), with the highest proportion attending junior high school (49.1%). Most participants came from complete families (82%), while only a small percentage were only children (17.9%) or left-behind children (28.6%). The study results showed that the most common health risk behaviors were mobile phone addiction (27.3%), picky eating (33.3%), insufficient sleep (56.5%), lack of physical activity (54.4%), and depressive symptoms (27.1%), whereas smoking (7.5%) and drinking (9.7%) were the least common.

### Patterns of adolescent health-risk behaviors

The fit indices for the two- to five-class models are presented in Table 5. The AIC, BIC, and adjusted BIC values decreased as the number of classes increased. Entropy increased from 0.647 for the two-class model to 0.729 for the three-class model and reached its highest value of 0.889 for the four-class model; however, it decreased markedly to 0.617 for the five-class model. Thus, entropy did not increase continuously with the number of classes. Although the five-class model had lower information criteria, the additional class accounted for only 3.6% of the sample and showed a behavioral pattern that substantially overlapped with the Multiple Health-Risk Behavior Group. The reduced entropy and limited substantive distinction of the additional class suggested possible class overextraction and classification instability. Therefore, the four-class model was retained because it provided the most appropriate balance among statistical fit, classification accuracy, parsimony, class size, and substantive interpretability.

**Table 5:** Fit indices of the latent class model for adolescent health risk behaviors.

#Classes	AIC	BIC	aBIC	Entropy	LMRLR*_(P)_*	BLRT*_(P)_*	Latent classes
2	102921.933	103061.431	103001.051	0.647	<0.001	<0.001	0.27562/0.72438
3	101759.406	101972.323	101880.165	0.729	<0.001	<0.001	0.25704/0.06706/0.67590
4	101380.188	101666.525	101542.588	0.889	<0.001	<0.001	0.06356/0.21785/0.45577/0.26282
5	101062.225	101421.982	101266.266	0.617	<0.001	<0.001	0.21618/0.05190/0.41992/0.03621/0.27580

AIC, Akaike information criterion; BIC, Bayesian information criterion; aBIC, sample-size adjusted BIC; LMRLR, the Lo–Mendell–Rubin likelihood ratio; BLRT, Bootstrap likelihood ratio test.

Four latent classes were identified: the Low-Risk Group (45.6%), the Physical Inactivity–Insufficient Sleep Group (21.8%), the Depressive Symptoms–Self-Harm–Picky Eating Group (26.3%), and the Multiple Health-Risk Behavior Group (6.4%). The Low-Risk Group was the largest in size, and the probability of occurrence for all health risk behaviors was below the average level of the total sample, with no dominant health-risk behaviors observed.

In the Physical Inactivity–Insufficient Sleep Group, three health-risk behaviors were found to have an occurrence probability higher than the average level, with physical inactivity and insufficient sleep as the core dominant behaviors (conditional probability >50%); among these, physical inactivity showed the highest conditional probability, representing the defining feature of this group.

The Depressive Symptoms–Self-Harm–Picky Eating Group was characterized by a total of seven health-risk behaviors with a higher-than-average probability of occurrence, with depressive symptoms, self-harm behavior and picky eating as the core dominant behaviors (conditional probability >50%); depressive symptoms had the highest conditional probability, being the most prominent feature of this group.

**Figure 1:**
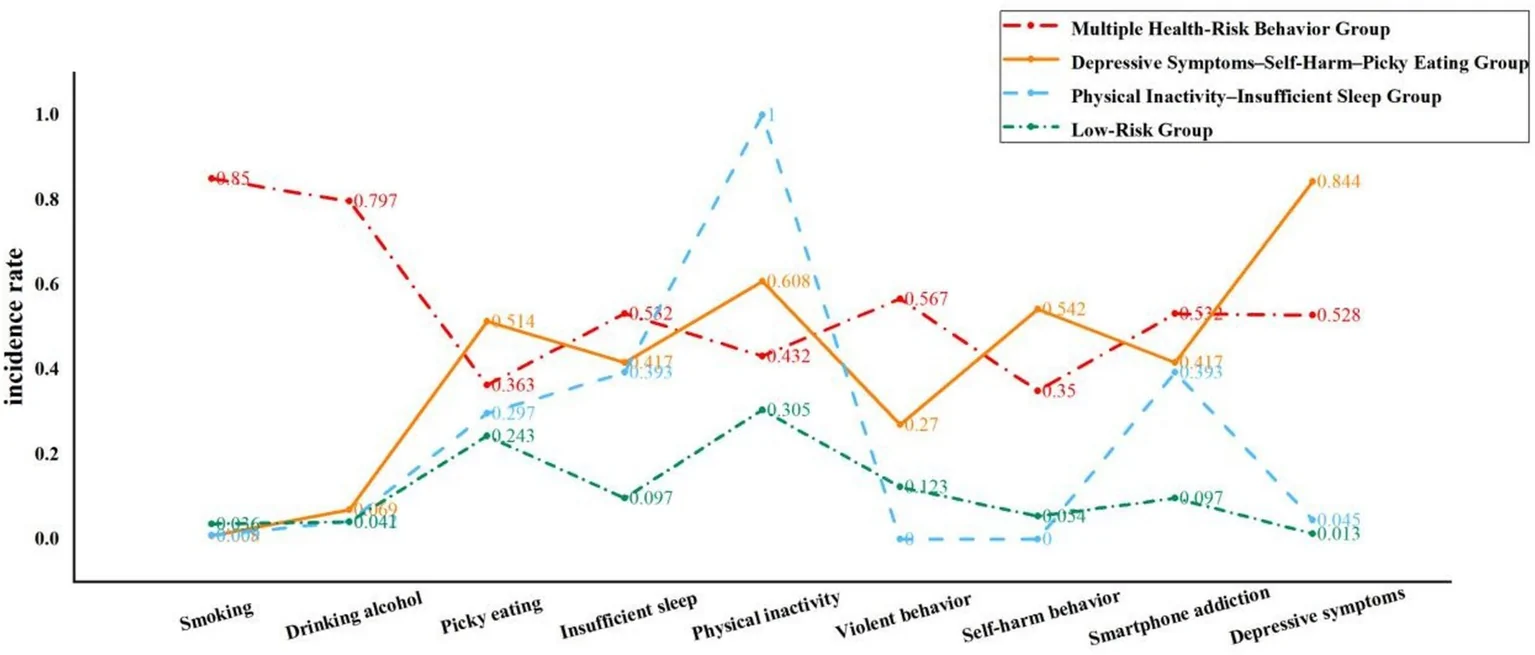
Occurrence of health-risk behaviors among different potential categories of adolescents in China.

In the Multiple Health-Risk Behavior Group, a total of eight health-risk behaviors showed an above-average occurrence probability, with smoking, drinking alcohol, violent behavior and smartphone addiction as the core dominant behaviors (conditional probability >50%); smoking and drinking alcohol were categorized as substance use, and violent behavior was defined as aggression in the class label, with smoking having the highest conditional probability. The clustering of multiple high-probability health-risk behaviors was the defining feature of this class. Figure 1 presents the conditional probability profiles of the four latent classes, and the corresponding numerical probabilities for all nine health-risk behaviors are provided in Supplementary Table S3.

### Stratified latent class analyses by educational stage

Additional latent class analyses were conducted separately among junior high school students (*n* = 5,605), senior high school students (*n* = 2,524), and university students (*n* = 3,278). Based on the model-fit indices, classification accuracy, class size, and substantive interpretability, the four-class solution was retained for all three educational-stage subgroups. Although the class proportions and class-specific conditional probabilities of health-risk behaviors varied across educational stages, four distinct patterns of health-risk behaviors were identified within each subgroup. Detailed model-fit indices and conditional probability profiles are presented in Supplementary Table S1; Supplementary Figure S1. The prevalence of all nine individual health-risk behaviors also differed significantly across educational stages (all *p* < 0.001) (Supplementary Table S2).

### Latent class feature analysis

Table 4 shows significant differences in sociodemographic and psychological characteristics across the four latent classes. Senior high school students had the highest proportions in the Depressive Symptoms–Self-Harm–Picky Eating Group and the Multiple Health-Risk Behavior Group, whereas university students had the highest proportion in the Physical Inactivity–Insufficient Sleep Group. The Low-Risk Group had the highest psychological resilience and self-control scores, while the Depressive Symptoms–Self-Harm–Picky Eating Group had the lowest resilience score and the Multiple Health-Risk Behavior Group had the lowest self-control score.

### The influence of psychological resilience and self-control on latent class membership

After multivariable adjustment, higher psychological resilience and self-control were associated with lower odds of membership in all three non-low-risk latent classes (Table 1). For psychological resilience, the ORs were 0.934 (95% CI: 0.925–0.942) for the Depressive Symptoms–Self-Harm–Picky Eating Group, 0.966 (95% CI: 0.957–0.976) for the Physical Inactivity–Insufficient Sleep Group, and 0.958 (95% CI: 0.945–0.972) for the Multiple Health-Risk Behavior Group. The corresponding ORs for self-control were 0.938 (95% CI: 0.932–0.943), 0.979 (95% CI: 0.973–0.986), and 0.912 (95% CI: 0.903–0.921), respectively.

Male sex was associated with lower odds of membership in the Physical Inactivity–Insufficient Sleep Group and the Depressive Symptoms–Self-Harm–Picky Eating Group, but with higher odds of membership in the Multiple Health-Risk Behavior Group. Rural residence was associated with lower odds of membership in all three non-low-risk classes. Educational stage was also significantly associated with class membership. Higher paternal education was associated only with the Physical Inactivity–Insufficient Sleep Group, whereas only-child status was associated only with the Multiple Health-Risk Behavior Group. Left-behind status and family structure were not independently associated with latent class membership after adjustment. Table 1 presents the adjusted main-effect estimates, whereas Table 2 reports the interaction terms. Psychological resilience interacted significantly with sex for all three non-low-risk classes. Its inverse associations with the Physical Inactivity–Insufficient Sleep Group (OR = 1.034, 95% CI: 1.014–1.054) and the Depressive Symptoms–Self-Harm–Picky Eating Group (OR = 1.033, 95% CI: 1.016–1.050) were weaker among males than among females, whereas its inverse association with the Multiple Health-Risk Behavior Group was slightly stronger among males (OR = 0.971, 95% CI: 0.943–0.999). Under the prespecified educational-stage coding, significant resilience-by-stage interactions were observed for the Depressive Symptoms–Self-Harm–Picky Eating Group (OR = 1.610, 95% CI: 1.246–2.080) and the Multiple Health-Risk Behavior Group (OR = 1.865, 95% CI: 1.156–3.008), while the self-control-by-stage interaction was significant only for the Multiple Health-Risk Behavior Group (OR = 0.979, 95% CI: 0.961–0.998). No significant interactions were found between self-control and sex or between either psychological factor and family structure.

## Discussion

This study explores the patterns of health-risk behaviors (HRBs) among Chinese adolescents through LCA, and examines the impact of psychological resilience and self-control on these patterns. The current study identified four distinct latent health risk behavior categories: the Low-Risk Group (45.6%), the Physical Inactivity–Insufficient Sleep Group (21.8%), the Depressive Symptoms–Self-Harm–Picky Eating Group (26.3%), and the Multiple Health-Risk Behavior Group (6.4%). It was also found that psychological resilience and self-control had significant effects on the latent class patterns. This survey provides a theoretical basis for current research and interventions on adolescent health risk behavior patterns in China.

The distributions of latent classes differed significantly across educational stages. Senior high school students had the highest proportions in the Depressive Symptoms–Self-Harm–Picky Eating Group and the Multiple Health-Risk Behavior Group, whereas university students had the highest proportion in the Physical Inactivity–Insufficient Sleep Group. Junior high school and university students had similar proportions in the Low-Risk Group.

Psychological resilience and self-control may be associated with lower involvement in health-risk behaviors through related but distinct psychological pathways. Psychological resilience may facilitate adaptive appraisal and emotion regulation when adolescents encounter academic, interpersonal, or family stress. Prospective evidence has shown that adaptive emotion-regulation strategies, particularly cognitive reappraisal, are associated with lower depressive symptoms among adolescents (50). In Chinese youth, resilience—particularly emotion regulation, interpersonal assistance, and family support—has also been found to mediate the association between self-harm and suicidal ideation (51). Self-control may operate more directly by helping individuals resist immediate temptations, develop health-promoting habits, and translate health-related intentions into sustained behavior (52). A recent meta-analysis further suggested that trait self-control is associated with health behavior partly through attitudes, perceived behavioral control, and behavioral intentions, with stronger effects for behaviors governed by impulsive processes (53). Taken together, psychological resilience may primarily support adaptive coping and recovery under stress, whereas self-control may help adolescents maintain goal-directed behavior. These complementary pathways may explain their protective associations with membership in the three non-low-risk latent classes.

This study is consistent with previous research (54, 55), Both domestic and international research results indicate that adolescents’ health-risk behaviors often do not occur in isolation, but coexist and are interrelated. A previous study has identified patterns of clustering of specific behaviors among adolescents, as well as patterns of health-risk behaviors among Chinese adolescents during the COVID-19 pandemic (56). Our study incorporates psychological variables to investigate their impact on potential patterns of health-risk behaviors in adolescents. Previous investigations usually found three (16) or four (56, 57) latent classes, Previous studies often used broad labels such as low-risk, medium-risk, and high-risk classes. Compared with previous literature, this study identified four potential categories and innovatively defined the names of these potential categories. Including the names of health risk behaviors in the definition of potential categories can help us better understand adolescent health risk behaviors and how to improve them through a peer-to-peer approach.

According to survey, currently smoking (58),physical inactivity (59) and depressive symptoms (60) are the most common health risk behaviors faced by Chinese adolescents. The Multiple Health-Risk Behavior Group was characterized by a higher representation of male adolescents high-risk behaviors are more common among male adolescents than female adolescents. This research finding is consistent with a study in China involving 7,663 students, which found that both the current smoking rate and the experimenter smoking rate were higher among boys than girls (61). In the “Multiple Health-Risk Behavior Group,” the highest proportion of high school students are those who smoke and are addicted to mobile phones, while among middle school students, the highest proportion is involved in deliberate harmful behaviors. It may be because high school students are socialized earlier than middle school students, making them more susceptible to negative influences and likely to become part of the smoking population (62). In addition, high school students face significant academic and psychological pressure, and they may choose to escape this pressure through the internet, leading to a higher reported rate of smartphone addiction (63), Since middle school students’ minds are not yet fully developed, they are easily impulsive, have relatively poor self-control, and are more likely to become involved in intentional harm behaviors (64). Combine the survey results to analyze and compare the incidence of depressive symptoms among American adolescents in recent years (13.1%) (65) and European adolescents (10.5%) (66). It has been found that the detection rate of depressive symptoms among adolescents in our country is higher than that in the United States and European countries. This difference may be related to factors such as statistical standards, cultural and social pressures, as well as awareness and healthcare-seeking rates. The incidence of depressive symptoms is higher in females than in males, suggesting that there is a gender difference in the occurrence of depressive symptoms (67). During puberty, girls face a series of physiological and psychological changes, among which fluctuations in estrogen may affect emotional stability, thereby increasing the risk of depression (68). In addition, girls are relatively more likely to encounter negative life events and have weaker coping abilities toward social events, which also increases their likelihood of developing depressive symptoms (69). Moderate to high levels of physical activity are important factors affecting mental health and can significantly improve the mental health of adolescents (70, 71). In the present study, 67.4% of university students were classified as physically inactive (Supplementary Table S2), which is consistent with previous findings (72). This phenomenon may be related to the reduction of physical education courses after college students enter university and the lack of exam-driven motivation, leading to a significant decrease in their participation motivation (73). In addition, we found that the level of physical activity among adolescents is negatively correlated with depressive symptoms (74), With the development of exercise psychology, physical activity has been widely recognized as a protective factor against anxiety and depressive symptoms (75). Therefore, encouraging college students to participate in physical activities may help increase physical activity levels and improve mental health, thereby reducing the risk of mental disorders. It is thus urgent and necessary to conduct in-depth investigations into the health risk behaviors of Chinese adolescents and their underlying causes in order to identify specific issues and explore possible solutions to reduce the occurrence of health risk behaviors among adolescents.

Research results show that the higher the level of psychological resilience, the less frequently health-risk behaviors occur. The low-risk group has the strongest psychological resilience. Adolescents with high resilience are able to regulate their emotions to cope with various difficulties, thereby avoiding the occurrence of harmful behaviors. Adolescents with higher resilience can regulate negative emotions in positive ways, reducing the vicious cycle of “stress-health risk behaviors.” This study verifies the positive role of psychological resilience, consistent with previous research (76), and also aligns with Fredrickson’s (77) broaden-and-build theory. The theory suggests that individuals with higher psychological resilience can more effectively mobilize positive emotional resources, buffering the development of depressive symptoms. Psychological resilience, as a positive psychological trait of individuals, can mobilize one’s own psychological resources, flexibly cope with setbacks or life stress, and recover from negative experiences or maintain normal functioning. Its advantage lies in preventing undesirable behaviors and gradually optimizing mental health (78), is an important internal force for individuals to cope with health-risk behaviors.

The research results show that the higher the level of self-control, the fewer health-risk behaviors occur. Individuals with a high incidence of health-risk behaviors often have low levels of self-control (79), Psychologists call it “insufficient self-control” (80). Individuals with strong self-control are able to reasonably regulate their emotions and behaviors; therefore, self-control is negatively correlated with health-risk behaviors. The “Multiple Health-Risk Behavior Group” has the weakest self-control. Smoking gives adolescents a sense of “pleasure” and is an externalized behavior. This requires adolescents to have strong resistance to temptation; those with lower self-control are more likely to succumb to temptation and take up smoking, increasing their likelihood of membership in the Multiple Health-Risk Behavior Group. The better adolescents are at self-control, the higher their subjective wellbeing (81). Previous studies have shown that individuals with high self-control tend to engage in healthy behaviors, thereby maintaining good physical and mental wellbeing (82), Individuals with low self-control are more prone to engage in health-risk behaviors, thereby affecting their physical and mental health (83).

These findings also have implications for the development of stage- and sex-sensitive prevention strategies. For junior high school students, school-based programs may emphasize emotional awareness, adaptive coping, impulse management, and parent–school support. For senior high school students, who showed relatively high proportions in the Depressive Symptoms–Self-Harm–Picky Eating Group and the Multiple Health-Risk Behavior Group, interventions may prioritize academic stress management, sleep education, early identification of depressive symptoms and self-harm, and resistance to substance-related peer influence. For university students, who were more frequently classified into the Physical Inactivity–Insufficient Sleep Group, programs may focus on autonomous time management, regular physical activity, sleep hygiene, and responsible smartphone use. Sex-specific components may also be beneficial. Prevention for male students may place greater emphasis on smoking, alcohol use, violence, and impulse control, whereas interventions for female students may give greater attention to depressive symptoms, self-harm, eating-related behaviors, and emotional coping. Rather than applying a uniform intervention to all students, schools could combine universal resilience- and self-control-building programs with targeted components based on educational stage, sex, and latent class characteristics.

All of the above findings indicate that it is necessary to use latent class regression to gain a comprehensive understanding of health-risk behaviors among Chinese adolescents and their influencing factors. If health-risk behaviors are defined solely based on a single, broad standard, it will be difficult to accurately classify specific health-risk behaviors, and the accuracy of the research results will be greatly reduced. Therefore, we redefined the potential categories based on health risk behaviors and further conducted an in-depth study on the distribution of different health risk behaviors among adolescents. In addition, we explored the relationship between adolescent psychological resilience and self-control and health risk behaviors from a psychological perspective, and found that high scores in psychological resilience and self-control were protective factors against membership in the Multiple Health-Risk Behavior Group, the Depressive Symptoms–Self-Harm–Picky Eating Group, and the Physical Inactivity–Insufficient Sleep Group. Self-control showed its strongest inverse association with membership in the Multiple Health-Risk Behavior Group, whereas psychological resilience showed its strongest inverse association with membership in the Depressive Symptoms–Self-Harm–Picky Eating Group.

## Limitations

This study has certain limitations. First, the questionnaire involved privacy-related psychological topics, and adolescents may have introduced self-report bias due to social expectations, self-enhancement, or defense mechanisms. In addition, the subsequent regression analyses were based on participants’ most likely latent class membership. Although the retained model showed good classification accuracy, this approach may not fully account for residual classification uncertainty. Second, this study used a cross-sectional design, making it difficult to determine the temporal order and causal relationships between variables. Third, the study did not involve intervention programs for psychological resilience and self-control, lacking exploration of the intrinsic mechanisms of such interventions. Fourth, although a multi-stage stratified cluster sampling method was used to recruit participants, sampling weights and class-level clustering were not incorporated into the statistical analyses. Therefore, the population-level generalizability and precision of the estimates should be interpreted with appropriate caution. In addition, the university subsample consisted predominantly of first- and second-year students, which may limit the generalizability of the findings to students in the later years of university. Nevertheless, the large sample size and broad geographic coverage provide valuable insights for developing preventive and intervention measures for adolescents’ health-risk behaviors. Therefore, to address these limitations, future research on adolescents’ health risk behaviors should adopt longitudinal study designs and include research on the intrinsic mechanisms of interventions.

## Conclusion

Despite its limitations, our study contributes to the exploration of potential latent class patterns of health-risk behaviors among Chinese adolescents and offers innovative research on the associations between psychological resilience, self-control, and adolescent health-risk behaviors. By integrating latent class profiling with psychological predictors, our findings provide an empirical foundation for precision prevention initiatives that simultaneously strengthen resilience and self-control skills among Chinese adolescents.

## Data Availability

The raw data supporting the conclusions of this article will be made available by the authors, without undue reservation.
