# Supplementary material for: Latent class profiles of health-risk behaviors and the protective roles of psychological resilience and self-control in Chinese adolescents
Source: Front Public Health. 2026 Aug 19;14:1908835. doi: 10.3389/fpubh.2026.1908835 (PMC13533798; doi:10.3389/fpubh.2026.1908835)
Supplement: Supplementary file 1 [file Data_Sheet_1.pdf]

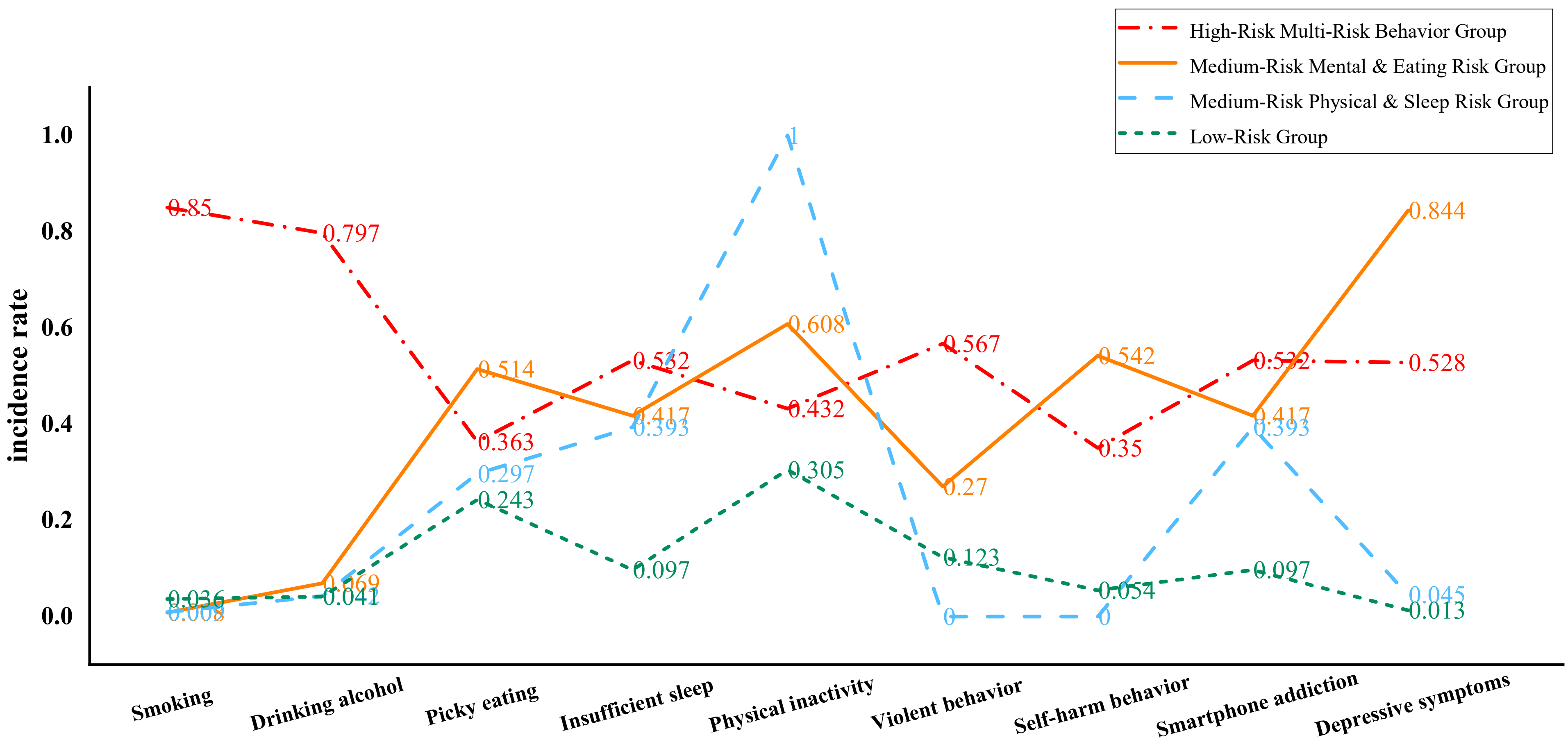

High-Risk Multi-Risk Behavior Group
Medium-Risk Mental & Eating Risk Group
Medium-Risk Physical & Sleep Risk Group
Low-Risk Group
incidence rate
1.0
0.8
0.6
0.4
0.2
0.0
Smoking
Drinking alcohol
Picky eating
Insufficient sleep
Physical inactivity
Violent behavior
Self-harm behavior
Smartphone addiction
Depressive symptoms
0.85
0.797
0.363
0.532
0.432
0.567
0.35
0.532
0.528
0.008
0.069
0.514
0.417
0.608
0.27
0.542
0.417
0.844
0.297
0.393
1
0
0
0.393
0.045
0.036
0.041
0.243
0.097
0.305
0.123
0.054
0.097
0.013
