# Supplementary material for: Latent class profiles of health-risk behaviors and the protective roles of psychological resilience and self-control in Chinese adolescents
Source: Front Public Health. 2026 Aug 19;14:1908835. doi: 10.3389/fpubh.2026.1908835 (PMC13533798; doi:10.3389/fpubh.2026.1908835)
Supplement: Supplementary file 2 [file Supplementary_file_1.docx]

| **#Classes** | **AIC** | **BIC** | **aBIC** | **Entropy** | **LMRLR*_（P）_*** | **BLRT*_(P)_*** | **latent classes** |
| --- | --- | --- | --- | --- | --- | --- | --- |
| 2 | 51414.442 | 51540.438 | 51480.062 | 0.649 | <0.001 | <0.001 | 0.32739/0.67261 |
| 3 | 50870.921 | 51063.232 | 50971.079 | 0.654 | <0.001 | <0.001 | 0.06548/0.30669/0.62783 |
| 4 | 50763.935 | 51022.561 | 50898.630 | 0.720 | 0.004 | <0.001 | 0.17734/0.06387/0.53631/0.22248 |
| 5 | 50708.465 | 51033.404 | 50877.697 | 0.661 | 0.001 | <0.001 | 0.03408/0.02926/0.23122/0.52810/0.17734 |

**Supplementary Table S1. Model-fit indices for educational-stage-stratified latent class analyses**

**Panel A. Junior high school students**

**Panel B. Senior high school students**

| **#Classes** | **AIC** | **BIC** | **aBIC** | **Entropy** | **LMRLR*_（P）_*** | **BLRT*_(P)_*** | **latent classes** |
| --- | --- | --- | --- | --- | --- | --- | --- |
| 2 | 23666.769 | 23777.607 | 23717.239 | 0.771 | <0.001 | <0.001 | 0.11569/0.88431 |
| 3 | 23190.833 | 23360.007 | 23267.867 | 0.780 | <0.001 | <0.001 | 0.56696/0.32647/0.10658 |
| 4 | 23088.462 | 23315.973 | 23192.060 | 0.655 | <0.001 | <0.001 | 0.09469/0.34786/0.05111/0.50634 |
| 5 | 23080.355 | 23366.201 | 23210.515 | 0.591 | 0.1367 | 0.030 | 0.05151/0.42195/0.34786/0.08558/0.09311 |

| **#Classes** | **AIC** | **BIC** | **aBIC** | **Entropy** | **LMRLR*_（P）_*** | **BLRT*_(P)_*** | **latent classes** |
| --- | --- | --- | --- | --- | --- | --- | --- |
| 2 | 22976.224 | 23092.028 | 23031.657 | 0.648 | 0.003 | <0.001 | 0.15497/0.84503 |
| 3 | 22686.375 | 22863.130 | 22770.984 | 0.775 | <0.001 | <0.001 | 0.12569/0.08542/0.78890 |
| 4 | 22536.882 | 22774.586 | 22650.666 | 0.821 | <0.001 | <0.001 | 0.07749/0.45577/0.08359/0.38316 |
| 5 | 22481.434 | 22780.089 | 22624.394 | 0.637 | 0.2198 | <0.001 | 0.00488/0.38316/0.41550/0.11165/0.08481 |

**Panel C. University students**

**Model selection**

Across the three educational-stage subgroups, the four-class solution was retained based on the overall balance of model fit, classification quality, class size, parsimony, and substantive interpretability. Among junior high school students, although the AIC and aBIC continued to decrease for the five-class model, the BIC increased, entropy decreased from 0.720 to 0.661, and the five-class solution generated two very small classes accounting for only 2.9% and 3.4% of the sample. Among senior high school students, the four-class model had lower BIC and aBIC values and higher entropy than the five-class model, and the LMR-LRT for the five-class solution was not statistically significant (P = 0.1367). Among university students, the four-class model showed higher entropy (0.821 vs. 0.637) and a lower BIC than the five-class model, while the LMR-LRT for the five-class solution was not statistically significant (P = 0.2198). In addition, the five-class model produced a very small class comprising only 0.5% of university students. Therefore, the four-class solution was considered the most parsimonious and substantively interpretable model for all three educational-stage subgroups.

**A. Junior High School Students**

**B. Senior High School Students**

**C. University Students**

**Supplementary Figure S1. Conditional Probability Profiles of Health-Risk Behaviors Across Latent Classes by Educational Stage**

**Note:** Four latent classes were retained within each educational-stage subgroup. Class labels were assigned according to the dominant conditional probability patterns of health-risk behaviors within each subgroup.

**Supplementary Table S2. Prevalence of Health-Risk Behaviors Across Educational Stages (n = 11407)**

| Health-risk behavior | Educational stage | | | *χ^2^* | *P* |
| --- | --- | --- | --- | --- | --- |
|  | Junior high school | Senior high school | University |  |  |
| Smoking | 317 (5.7) | 247 (9.8) | 286 (8.7) | 53.865 | <0.001 |
| Drinking alcohol | 390 (7.0) | 290 (11.5) | 424 (12.9) | 96.684 | <0.001 |
| Smartphone addiction | 1260 (22.5) | 681（27.0） | 1176 (35.9) | 187.091 | <0.001 |
| Picky eating | 2087 (37.2) | 1161 (46.0） | 556 (17.0) | 615.818 | <0.001 |
| Insufficient sleep | 3037 (54.2) | 1875 (74.3) | 1528 (46.6) | 467.375 | <0.001 |
| Physical inactivity | 2598 (46.4) | 1402 (55.5) | 2208 (67.4) | 369.620 | <0.001 |
| Depressive symptoms | 1806 (32.2) | 984 (39.0) | 302 (9.2) | 785.656 | <0.001 |
| Violent behavior | 1432 (25.5) | 371 (14.7) | 56 (1.7) | 867.851 | <0.001 |
| Self-harm behavior | 1537 (27.4) | 550 (21.8) | 74 (2.3) | 870.072 | <0.001 |

**Note:** Values are presented as unweighted n (%). Differences in the prevalence of health-risk behaviors across educational stages were examined using the *χ²* test.

**Supplementary Table S3. Class-Specific Conditional Probabilities (%) of the Nine Health-Risk Behaviors in the Full-Sample Four-Class Model**

| **Health-risk behavior** | **Multiple Health-Risk Behavior Group** | **Physical Inactivity–Insufficient Sleep Group** | **Low-Risk Group** | **Depressive Symptoms–Self-Harm–Picky Eating Group** |
| --- | --- | --- | --- | --- |
| **Smoking** | 84.97 | 0.89 | 3.62 | 0.80 |
| **Drinking alcohol** | 79.72 | 4.23 | 4.14 | 6.87 |
| **Picky eating** | 36.28 | 29.70 | 24.27 | 51.40 |
| **Insufficient sleep** | 64.14 | 80.72 | 30.76 | 79.05 |
| **Physical inactivity** | 43.17 | 100.00 | 30.54 | 60.77 |
| **Violent behavior** | 56.69 | 0.00 | 12.27 | 27.02 |
| **Self-harm behavior** | 35.03 | 0.00 | 5.42 | 54.20 |
| **Smartphone addiction** | 53.24 | 39.32 | 9.67 | 41.73 |
| **Depressive symptoms** | 52.83 | 4.51 | 1.29 | 84.39 |

**Note:** Values represent the estimated conditional probabilities, expressed as percentages, of endorsing each health-risk behavior within each latent class.
